# Robotic transesophageal echocardiography: system design and deep learning-based kinematic modeling

**DOI:** 10.3389/frobt.2025.1705142

**Published:** 2026-01-27

**Authors:** Seyed MohammadReza Sajadi, Abbas Tariverdi, Henrik Brun, Ole Jakob Elle, Kim Mathiassen

**Affiliations:** 1 Department of Informatics, University of Oslo, Oslo, Norway; 2 Department of Physics, University of Oslo, Oslo, Norway; 3 The Intervention Center, Oslo University Hospital, Oslo, Norway; 4 Department for Pediatric Cardiology, Oslo University Hospital, Oslo, Norway; 5 Department of Technology Systems, University of Oslo, Oslo, Norway

**Keywords:** robotic transesophageal echocardiography, deep learning kinematic modeling, LSTM, robotic-assisted echocardiography, synchronized robotic subsystems, cable-driven mechanism, continuum mechanism

## Abstract

**Introduction:**

This paper presents a robotic transesophageal echocardiography (TEE) system that replicates all essential degrees of freedom available in manual TEE procedures. The developed robotic system advances dual-subsystem architectures through enhanced mechanical design and deep learning-based kinematic modeling.

**Methods:**

Building upon previous designs that manipulate the TEE probe from both handle and gastroscope tube, our system integrates with a teleoperated UR5 manipulator to accommodate both supine and left lateral decubitus patient positions, addressing the full spectrum of clinical TEE procedures. The system features 6 DOF at the probe handle and 2 DOF at the gastroscope tube. Together, these create optimal gastroscope tube geometry, minimizing cable tension asymmetry and friction-induced nonlinearities inherent in cable-driven mechanisms. The primary contribution is a data-driven kinematic model using recurrent neural networks with LSTM units that overcomes fundamental limitations of analytical approaches for continuum manipulators. Trained on 42,000 synchronized pose-command pairs collected across three gastroscope tube configurations (0°, 45°, 90° bends), the model effectively captures dead zones, hysteresis, and coupling effects between steering mechanisms.

**Results:**

Experimental validation demonstrates strong position tracking across all three gastroscope tube configurations. The model achieves RMSE of 1.267 mm for the 0° configuration, 1.209 mm for the 45° configuration, and 1.194 mm for the 90° configuration. Mean orientation errors are 7.064° at 0°, 8.503° at 45°, and 4.947° at the clinically critical 90° configuration. The model exhibits coordinate frame independence with only 0.06 mm RMSE difference between original and rotated datasets. This confirms true kinematic learning rather than coordinate-specific patterns. With 1.8 ms inference time, the system achieves real-time performance essential for clinical deployment.

**Discussion:**

This integration of robotic system design with deep learning establishes a foundation for semi-autonomous TEE systems. The developed system can support both diagnostic TEE examinations and TEE-guided structural heart interventions.

## 1 Introduction

Ultrasound imaging is a safe and non-invasive tool that can penetrate deep into soft tissues and provides spatial and temporal resolution without harmful ionizing radiation. It is an indispensable tool for echocardiographers due to its non-invasiveness, real-time imaging capabilities, cost-effectiveness, and ease of use (Klibanov and Hossack, 2015). Transesophageal echocardiography (TEE) is a high-resolution ultrasound imaging technique where the transducer is positioned in the esophagus, close to the heart, during image acquisition. The esophagus’s proximity to the heart’s posteromedial side, without intervening lung or bone, allows TEE to use high-frequency imaging transducers, resulting in superior spatial resolution (Patel et al., 2022).

TEE provides exceptional visualization of cardiac structures, particularly cardiac valves, offering enhanced real-time three-dimensional (RT-3D) imaging for guiding ultrasound image-guided interventions. This technique is especially valuable for navigating catheters and the placement of devices such as occluders for atrial and ventricular septal defects, left atrial appendage or paravalvular leaks (Basman et al., 2017). TEE is commonly used for paravalvular leak closure procedures due to its ability to provide a clear, close-up view of the aortic valve and surrounding structures. During procedures, it offers real-time guidance that enables proper positioning and deployment of transcatheter aortic valves. Furthermore, this imaging approach is mandatory for Transcatheter Edge-to-Edge Repair (TEER) of the mitral valve, providing essential visualization and guidance throughout the intervention (Giblett et al., 2019).

TEE is the gold standard for diagnosing and treating valvular heart disease, guiding catheter-based interventions and providing intraoperative evaluation during surgery (Tsai, 2013). Despite its critical role, interventional echocardiography (IE) specialists face significant challenges that have only recently gained widespread recognition with the development of standardized training curricula by the American and European societies to ensure safety and effectiveness in structural heart disease (SHD) interventions (Agricola et al., 2021; Little et al., 2023). However, using hand-held TEE probes during prolonged fluoroscopy-guided procedures exposes echocardiographers to high levels of ionizing radiation—studies show that IE specialists experience radiation exposure 3 to 12 times higher than interventional cardiologists, with up to 10 percent of radiation penetrating protective shields (McNamara et al., 2022; Crowhurst et al., 2018). The use of hand-held TEE probes also requires echocardiographers to maintain ergonomically challenging postures for extended periods. Additionally, TEE requires specialized skills, and the quality of ultrasound images depends entirely on the operator’s proficiency (Agricola et al., 2021; Little et al., 2023). The shortage of echocardiographers, even in central hospitals, combined with the increased demand for cardiac interventions for structural and valvular heart disease, presents a significant challenge for healthcare systems (Sajadi et al., 2025).

These challenges can be effectively addressed by developing robotic-assisted TEE systems that closely replicate the manual use of hand-held TEE probes by echocardiographers. This system should include all the essential degrees of freedom found in hand-held TEE probes. Consequently, it can offer significant advantages: shielding echocardiographers from radiation exposure, saving the time of experienced echocardiographers by reducing unnecessary relocations and travel, providing the possibility of remote mentoring and training, and automating probe manipulation to prevent the need for maintaining ergonomically challenging postures for extended periods.

While the robotic-assisted system for TTE is a well-researched subject (Mathiassen et al., 2016; Sajadi et al., 2022a; Sajadi et al., 2025; Solvin et al., 2023), The robotization of TEE probes is a relatively new field. The first attempt at TEE probe robotization was pioneered by S. Wang et al., who developed a 4 degree-of-freedom (DOF) robotic system for the X7-2t Philips TEE probe (Wang et al., 2016a). This system was designed to manipulate the TEE probe from its handle, using a passive guiding mechanism with articulation arms to guide the gastroscope tube (Wang et al., 2016a; Wang et al., 2016b). Xie et al. (2023) developed an upgraded version, also aiming to robotize the handle of a TEE probe with 4 DOF. They implemented a passive translation guidance mechanism to guide the gastroscope tube. Our preliminary design follows these trends by robotizing the handle of a TEE probe. In addition to the 4 DOF, our previous study also included the ability to manipulate the imaging plane, but it did not control the gastroscope tube (Sajadi et al., 2022b).

Recently, Schewel et al. introduced a remote-control robotic (RCR) system for TEE consisting of two components that manipulate both the TEE probe handle and gastroscope tube (Schewel et al., 2024). Their ROB’E system provides 5 degrees of freedom through a ROB’E Base unit that controls four DOFs at the handle (anteflexion/retroflexion, lateral flexion, multiplane rotation, and handle tilt) and a ROB’E Guide unit positioned near the patient’s mouth that manages probe advance/withdraw and axial rotation. The system demonstrated successful manipulation and position reproducibility through bench testing and evaluation in a Blue Phantom nonbeating heart simulator (Schewel et al., 2024), followed by first-in-human testing in five patients that established clinical feasibility with successful acquisition of standard TEE views (Schewel et al., 2025). This work represented a significant contribution to robotic TEE by establishing the feasibility of dual-component manipulation through validation from bench testing to first-in-human clinical trials.

However, certain design considerations remain for broader clinical implementation. To the best of our knowledge, the ROB’E system mounts the gastroscope tube control mechanism (ROB’E Guide) at the head end of the operating table. While the Guide’s position along the table rail can be adjusted, its orientation remains fixed for vertical probe insertion. This configuration is well-suited for supine positioning, where the probe is inserted vertically into the patient’s mouth with the patient lying flat on their back. However, this fixed configuration presents challenges for left lateral decubitus positioning, where the probe insertion angle and spatial relationship differ substantially from supine positioning.

According to established TEE practice, awake patients are usually intubated in the lateral decubitus position to facilitate drainage of saliva (Flachskampf et al., 2001). More specifically, in elective procedures performed in awake, fasting patients undergoing TEE with conscious sedation, patients are placed in the left lateral decubitus position to minimize the risk of aspiration (Côté and Denault, 2008). This positioning is standard practice in echocardiography laboratories where, as specified in the ASE/SCA guidelines, “the patient is typically placed in the left lateral decubitus position” and “the echocardiographer stands facing the patient on the left-hand side of the stretcher” (Hahn et al., 2013). While studies have demonstrated that TEE can be successfully performed in either left or right lateral decubitus positions with comparable image quality for most cardiac structures (Stoddard et al., 1993), left lateral decubitus remains the conventional standard for awake patients undergoing diagnostic procedures, where the probe insertion angle differs substantially from the vertical insertion path used in supine positioning.

Another significant challenge in TEE probe robotization is modeling the probe’s cable-driven mechanism. Various approaches to kinematic modeling of continuum manipulators have been reported in the literature (Webster III and Jones, 2010). Some methods use modified Denavit-Hartenberg (DH) parameters (Jones and Walker, 2006), while others rely on geometrical principles, such as the constant curvature and piecewise constant curvature models (Wang et al., 2016a). The constant curvature model is the basis for most kinematic studies of continuum robots (Loschak et al., 2013; Wang et al., 2016a; Webster III and Jones, 2010). This model assumes that plastic torsion in active bending is negligible and that bending occurs on a plane with a constant radius of curvature (Wang et al., 2016a). The main challenge in kinematic modeling of the TEE probe lies in the complex nonlinear behavior of its cable-driven mechanism, as demonstrated in our preliminary work (Sajadi et al., 2022b). This mechanism exhibits multiple dead zones where it fails to respond to robotic commands, introducing significant nonlinearities into the model. Another significant source of these nonlinearities is the variable friction between the driving cables and the inner surface of the gastroscope tube, which occurs when the flexible tube undergoes different curvatures during manipulation.

Building upon the validated system established by Schewel et al. (2024), Schewel et al. (2025), this paper makes two primary contributions addressing shortcomings in both robotic system design and kinematic modeling: hardware adaptations for broader clinical implementation and a deep learning-based kinematic modeling approach that represents the next technological step toward semi-autonomous robotic TEE through the integration of deep learning with robotic TEE systems.

- Robotic System Adaptation for Broader Clinical Implementation: We adapted the dual-subsystem architecture validated by Schewel et al. (2024), Schewel et al. (2025) to address expanded clinical applicability. While the ROB’E system is well-suited for supine positioning, it presents challenges for left lateral decubitus positioning, which remains the conventional standard for awake patients undergoing diagnostic procedures with conscious sedation. Our system incorporates all DOFs from the ROB’E design while introducing three key enhancements: (1) integration with a teleoperated UR5 collaborative manipulator that accommodates both supine and left lateral decubitus patient positioning, (2) rail-mounted advance/withdraw capability that replicates standard manual TEE technique with synchronized dual-point manipulation, and (3) an additional handle inclination DOF that reduces gastroscope tube bending and minimizes friction-induced nonlinearities. The resulting system features a 6-DOF robotic holder subsystem that manipulates the probe handle and a 2-DOF add-on subsystem mounted on the UR5 manipulator that controls the gastroscope tube. Redundant DOFs in both subsystems are precisely synchronized using a leader-follower hardware architecture to ensure gastroscope tube geometry stability.
- Deep Learning-Based Kinematic Modeling: The dual-point manipulation strategy stabilizes gastroscope tube geometry, creating predictable dead zone behavior suitable for data-driven modeling. We developed an LSTM-based kinematic model trained on 42,000 synchronized pose-command pairs across three gastroscope tube configurations (0°, 45°, 90° bends). The data were collected using an optical tracking system and represent clinical scenarios from initial insertion to transgastric views. To ensure robustness and coordinate frame independence, the model was trained and evaluated on both original and coordinate-transformed datasets, demonstrating consistent performance across different reference frames for deployment in varying clinical setups. This deep learning integration addresses the fundamental kinematic modeling challenges of the TEE probe’s cable-driven continuum mechanism. It enables accurate real-time prediction of transducer position and orientation despite the complex nonlinearities, dead zones, and friction-induced variations inherent in such systems.

These contributions enable broader clinical implementation and provide accurate kinematic modeling essential for advanced control strategies. Our robotic platform operates as a standalone unit without requiring human presence, similar to Schewel’s system, but accommodates both supine and left lateral decubitus positioning. The system can function as a follower robot in teleoperation setups and provides the foundation for future semi-autonomous capabilities in image-guided cardiac interventions.

## 2 System design

The design strategy presented in this paper involves developing a robotic system that interfaces with a commercially available GE 6Vt-D TEE probe, providing comprehensive control over both the probe handle and the gastroscope tube. This approach leverages the probe’s existing FDA-approved design and widespread clinical adoption. By building upon an established medical device, we inherently address critical requirements such as biocompatibility, dimensional constraints, and sterilization protocols, while substantially reducing development time and prototyping costs. The conceptual design phase prioritized alignment with clinical workflows and requirements. Our primary objective was to faithfully replicate how echocardiographers manually manipulate the TEE probe, ensuring the robotic system remains clinically relevant and compatible with standard TEE procedures.

### 2.1 Clinical requirement - design specification

The GE 6Vt-D TEE probe operates within a frequency range of 3–8 MHz, features a 90-degree field of view, and enables 180 degrees of electronic rotation. The probe comprises four main components: an electrical connection, a handle, a gastroscope tube, and a continuum mechanism. Two control knobs on the left side of the handle adjust the imaging plane within the ultrasound volume, while two steering wheels on the handle operate the bending mechanism. These wheels drive pull-wires that extend through the entire length of the gastroscope tube to the continuum mechanism, where the ultrasound transducer is housed.

**FIGURE 1:**
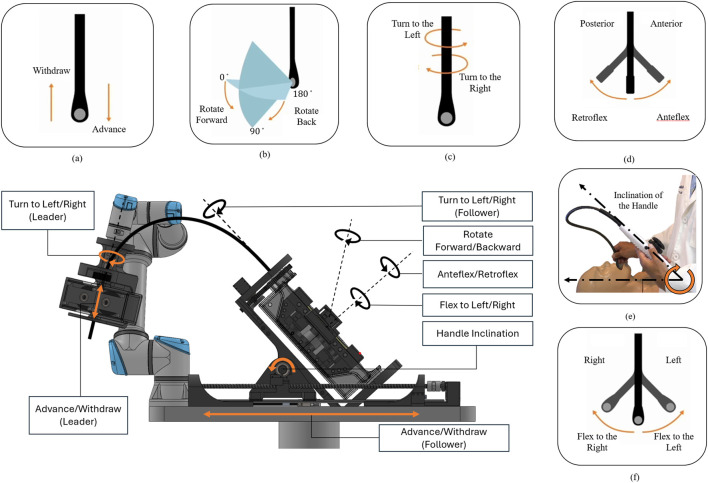
Clinical terminology for TEE probe manipulation and conceptual design of the developed robotic system in this paper. **(a–f)** Standard TEE probe movements: **(a)** advance/withdraw, **(b)** multiplane angle rotation (0°–180°), **(c)** left/right turning, **(d)** anteflexion/retroflexion, **(e)** handle inclination, and **(f)** lateral flexion. The center panel shows the developed robotic system featuring synchronized leader-follower subsystems: a 6-DOF robotic holder controlling the handle (follower for advance/withdraw and turn to Left/Right) and a 2-DOF add-on subsystem mounted on a UR5 manipulator controlling the gastroscope tube (leader for advance/withdraw and turn to Left/Right).

During manual TEE examinations, echocardiographers manipulate the probe using both the handle and the gastroscope tube to position the transducer at specific locations within the esophagus, in close proximity to cardiac structures. Cross-sectional ultrasound images are obtained by adjusting the multiplane angle via the control knobs. The standard terminology for describing TEE probe manipulation during image acquisition is illustrated in Figure 1. Based on these clinical requirements, our robotic system incorporates 6 degrees of freedom at the handle and 2 degrees of freedom along the gastroscope tube to achieve full transducer control.

The robotic holder subsystem provides advance/withdraw motion ranging from 20–25 cm minimum to 45–50 cm maximum, left/right turning through 180°, anteflexion up to 120° and retroflexion up to 40°, lateral flexion up to 40° in each direction, multiplane rotation up to 120°, and handle inclination up to 70°. The add-on robotic subsystem shares the same specifications for advance/withdraw (20–50 cm) and turning (180°) movements, with these redundant DOFs synchronized between both subsystems. These parameters were derived from the GE 6Vt-D technical specifications and validated against standard transducer positions and multiplane angles reported in established TEE guidelines Shanewise et al. (1999); Hahn et al. (2013); Jaidka et al. (2019).

### 2.2 Conceptual design

The robotic system developed in this paper builds upon the concept of dual-component TEE architecture validated by Schewel et al. (2024), Schewel et al. (2025). We extend this proven design to accommodate both supine and left lateral decubitus patient positioning, addressing broader clinical requirements for TEE examination. By adding two additional DOFs and integrating with the UR5 manipulator, our system provides enhanced flexibility and more predictable mechanical behavior for deep learning modeling integration.

The ROB’E system comprises two subsystems: the ROB’E Base and the ROB’E Guide. The ROB’E Base controls 4 DOF at the probe handle (lateral flexion, anteflexion/retroflexion, rotate forward/backward, and turn left/right). The ROB’E Guide controls 2 DOF at the gastroscope tube (advance/withdraw and turn left/right), with the turn left/right motion synchronized between both components.

Two key limitations affect this design. First, the ROB’E Guide remains fixed at the head of the operating table, as stated in their paper (Schewel et al., 2024). While this configuration suits supine positioning during interventional procedures, it presents constraints for left lateral decubitus positioning commonly required in diagnostic TEE examinations. Second, the fixed-position architecture induces a sharp U-shaped configuration in the gastroscope tube between the Guide and Base subsystems. This sharp curvature increases friction within the cable-driven mechanism, creates unpredictable force transmission, and enlarges dead zones during anteflexion/retroflexion and lateral flexion movements.

As illustrated in Figure 1, the developed robotic system encompasses all DOF provided by the ROB’E system while addressing these limitations. This is achieved through additional degrees of freedom and integration with the teleoperated UR5 system. Unlike single-point manipulation approaches (Xie et al., 2023; Wang et al., 2016a; Wang et al., 2016b; Sajadi et al., 2022b) that control the probe solely from the handle, our dual-subsystem architecture manipulates both the probe handle and gastroscope tube, closely replicating how echocardiographers naturally perform clinical procedures. The robotic system comprises two synchronized subsystems. The first is a 6-DOF robotic holder that manipulates the probe handle to precisely replicate manual TEE techniques documented in clinical guidelines (Shanewise et al., 1999; Hahn et al., 2013; Jaidka et al., 2019). The second is a 2-DOF add-on subsystem mounted on the UR5 end-effector, controlling advance/withdraw and turn left/right motions at the gastroscope tube. This architecture maintains all advantages of the ROB’E system while providing enhanced mechanical flexibility through the incorporation of two additional DOFs and UR5 robotic manipulator integration.

Our system introduces two principal enhancements over the previous architecture:

1. Robotic Add-on Subsystem Positioning Through UR5 Integration: The developed system integrates with a previously validated teleoperated UR5 collaborative manipulator (Sajadi et al., 2025; Mathiassen et al., 2016), demonstrated for both short-distance and long-distance teleoperation. Unlike Schewel’s fixed ROB’E Guide configuration, our add-on subsystem mounts to the UR5 end-effector, enabling dynamic repositioning to accommodate both supine and left lateral decubitus patient orientations. This adaptability can be achieved through either the UR5’s built-in free-drive mode or via the teleoperation framework previously validated for remote ultrasound procedures (Sajadi et al., 2025; Mathiassen et al., 2016). This configuration not only eliminates positioning constraints but also establishes the framework for future fully robotic probe insertion, advancing beyond current manual insertion requirements.
2. Optimized Gastroscope Tube Geometry: To address the sharp U-shaped deflection of the gastroscope tube, we implemented the standard manual TEE holding technique where the probe handle maintains an angular offset from the patient’s body axis. Our system incorporates two critical DOFs absent in the ROB’E design. First, a handle inclination DOF provides smooth angular adjustment ranging from 0° to 70° between the probe handle and horizontal plane, unlike Schewel’s fixed 90° configuration or other systems fixed at 0°. Second, a rail-mounted advance/withdraw mechanism operates at the handle position. This combination enables a more gradual gastroscope tube curvature, eliminating the sharp U-shaped deflection characteristic of the previous systems. Sharp U-shaped configurations create asymmetric cable tension distribution, where outer radius cables experience higher tension than inner radius cables. This asymmetry exacerbates hysteresis and backlash during motion reversal in both the small and large wheels of the handle. Our gentle bend profile significantly reduces cable tension asymmetry, minimizing friction-induced nonlinearities and dead zones. This mechanical optimization not only creates more predictable system behavior for deep learning integration but also reduces mechanical stress on the expensive TEE probe during manipulation.

The synchronized control architecture maintains coordinated motion between both subsystems throughout the manipulation envelope. Our 6-DOF robotic holder combined with the 2-DOF add-on subsystem delivers comprehensive probe control while the UR5 integration enables seamless adaptation to varying patient positions. The additional DOFs create optimal gastroscope tube geometry that minimizes mechanical nonlinearities, establishing a robust platform for deep learning integration. This enhanced mechanical design provides the flexibility, precision, and predictability essential for advancing automated TEE procedures in diverse clinical scenarios, from diagnostic examinations to complex structural heart interventions.

### 2.3 Engineering design and prototyping

**FIGURE 2:**
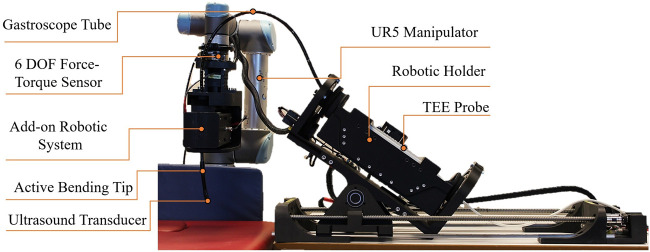
Prototype of the robotic TEE system featuring the 6-DOF robotic holder, 2-DOF add-on subsystem mounted on UR5 manipulator, and integrated TEE probe. This Prototype is Based on the Conceptual Design and Meets the Clinical Requirements.

Figure 2 shows the prototype of the robotic system developed in this paper. This prototype precisely aligns with the conceptual design and meets clinical requirements. It integrates robotic holder subsystems, the UR5 manipulator, and the add-on robotic subsystem. The engineering design process for each degree of freedom is outlined as follows:

#### 2.3.1 Advance/withdraw

Advancing the transducer involves pushing it through the esophagus toward the stomach, while withdrawal refers to retracting it in the opposite direction. This degree of freedom requires synchronized manipulation of both the handle and the gastroscope tube.

##### 2.3.1.1 Advance/withdraw in the add-on robotic subsystem

**FIGURE 3:**
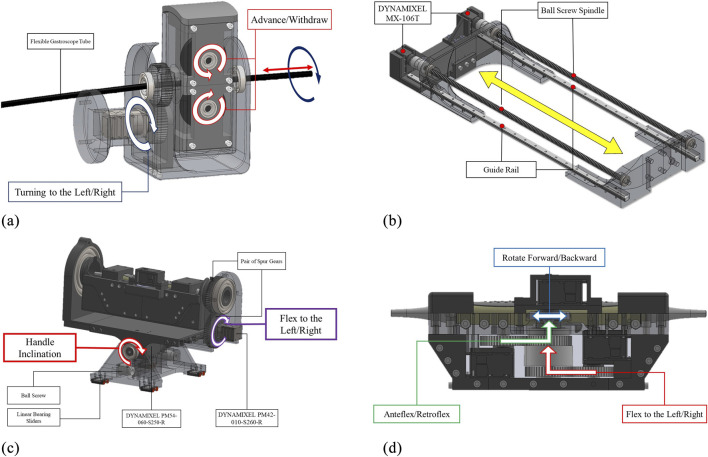
Engineering design of the developed robotic system in this paper: **(a)** Add-on subsystem with leader controls for advance/withdraw and left/right turning mechanisms, **(b)** Robotic holder’s follower advance/withdraw mechanism synchronized with the add-on subsystem, **(c)** Holder subsystem featuring handle inclination control and follwer left/right turning mechanism, and **(d)** Holder subsystem’s probe manipulation controls including anteflexion/retroflexion, lateral flexion, and multiplane rotation. Redundant DOFs between subsystems are synchronized to ensure identical coordinated motions.

This serves as the leader DoF, providing precise control over advancing and withdrawing movements. As shown in Figure 3a, a double-drive pulley mechanism securely grips the flexible gastroscope tube between two pulleys with radius $r_{p}=35$ mm. Each pulley is powered by a DYNAMIXEL MX-106R servomotor delivering 8.4 N ⋅ m of stall torque, providing sufficient force for reliable tube manipulation.

##### 2.3.1.2 Advance/withdraw in the robotic holder subsystem

Due to the passive nature of the flexible gastroscope tube, this DoF operates as the follower to maintain consistent tube curvature. As illustrated in Figure 3b, two DYNAMIXEL MX-106T servomotors drive ball screw mechanisms with linear sliders. These motors operate in synchronized dual mode via a 3-wire connection. The ball screw mechanism features a 700 mm stroke and 10 mm lead, achieving a resolution of 0.002 mm per pulse given the servomotor’s 4096 pulses per revolution.

##### 2.3.1.3 Kinematic mapping between subsystems

The different mechanisms used for this DOF in the two subsystems necessitate differential kinematic mapping to ensure synchronized motion from a single robotic command. For the leader (add-on subsystem) with pulley radius $r_{p}=35$ mm, the linear displacement of gastroscope tube is:

$$x_{\mathrm{tube}}=r_{p}\cdot \theta_{L}$$ (1)

where $\theta_{L}$ is the angular displacement of the motors in add-on subsystem in radians.

For the follower (holder subsystem) with ball screw lead p=10 mm, the linear displacement of the probe handle is:

$$x_{\mathrm{holder}}=\frac{p}{2\pi }\cdot \theta_{F}$$ (2)

where $\theta_{F}$ is the angular displacement of the motors in robotic holder.

To maintain synchronized linear motion $\left(x_{\mathrm{tube}}=x_{\mathrm{holder}}\right)$ , the angular displacement relationship becomes:

$$\theta_{F}=\frac{2\pi \cdot r_{p}}{p}\cdot \theta_{L}=7\pi \cdot \theta_{L}\approx 22\cdot \theta_{L}$$ (3)

To achieve equal displacement in both subsystems, the motors must be driven with this differential mapping. This 1:22 kinematic mapping ensures that for every radian of rotation in the leader subsystem, the follower subsystem rotates 7π radians, maintaining coordinated advance/withdraw motion while compensating for the different mechanical transmissions.d.

#### 2.3.2 Turn to the left/right

Rotating the transducer clockwise within the esophagus towards the patient’s right side is termed “turn to the right,” while rotating it counterclockwise is referred to as “turn to the left.” This degree of freedom requires synchronized manipulation from both the handle and the gastroscope tube.

##### 2.3.2.1 Turn to left/right in the add-on robotic subsystem

This degree of freedom (DoF) in the add-on robotic subsystem is designated as the leader DoF, enabling precise control of left and right movements. For this DoF, a DYNAMIXEL PM42-010-S260-R servomotor, coupled with a pair of spur gears with a 1:1 power ratio, is used, as shown in Figure 3a.

##### 2.3.2.2 Turn to left/right in the robotic holder subsystem

The corresponding DoF in the robotic holder subsystem is designated as the follower DoF. It employs the same servomotor and mechanism as the leader DoF, as illustrated in Figure 3c. Both the leader and follower DoFs are synchronized and controlled by the same robotic command.

#### 2.3.3 Anteflex/retroflex

Anteflexion refers to bending the probe tip anteriorly, while retroflexion involves posterior bending. The large steering wheel controls this DoF through a DYNAMIXEL MX-106T servomotor coupled with 1:1 ratio spur gears, as shown in Figure 3d.

#### 2.3.4 Flex to left/right

Lateral flexion enables bending the probe tip toward the patient’s right or left side. The small steering wheel controls this movement via a DYNAMIXEL MX-106T servomotor with 1:1 ratio spur gears, as depicted in Figure 3d.

#### 2.3.5 Rotate forward/backward

This DoF controls the multiplane imaging angle from 0° to 180°. Forward rotation increases the angle toward 180°, while backward rotation returns it toward 0°. Two control knobs on the probe handle are directly driven by a DYNAMIXEL MX-64T servomotor, as shown in Figure 3d.

#### 2.3.6 Handle inclination

Proper handle inclination prevents probe damage during advance/withdraw movements (Jaidka et al., 2019). This critical DoF accommodates the required angular positioning between the handle and the patient’s body. Given the substantial mechanical load, a high-torque DYNAMIXEL PM54-060-S250-R servomotor with dual bearing support provides the necessary stability and precision, as illustrated in Figure 3c.

### 2.4 Control architecture and electrical system design

Due to the absence of commercially available leader robots suitable for TEE manipulation, we developed a custom teleoperation architecture using a LabVIEW interface. This centralized control system currently coordinates both robotic subsystems through a unified user interface for local operation. While our current implementation focuses on local control, the LabVIEW platform’s built-in network capabilities provide a straightforward path for extending the system to support remote teleoperation. Our previous validation of 5G networks for long-distance ultrasound teleoperation (Sajadi et al., 2025) demonstrates the feasibility of extending this system for remote operation, which could address echocardiographer shortages and enable TEE services in remote areas.

In this control architecture, “leader-follower” refers specifically to the synchronization of redundant DOFs between subsystems (advance/withdraw and turn left/right). It is important to clarify that the add-on robotic subsystem does not control all of the robotic holder’s DOFs. Only these two redundant DOFs are synchronized in a leader-follower configuration to enhance manipulation precision and maintain stable gastroscope tube geometry. The add-on subsystem acts as the “leader” for these synchronized motions, receiving commands from the LabVIEW interface and providing encoder feedback as the reference for the robotic holder subsystem (follower) to maintain coordinated movement through the kinematic mappings. For advance/withdraw, a 1:22 differential mapping ensures equal linear displacement despite different mechanical transmissions (Section 2.3.1.3), while turn left/right maintains a 1:1 ratio (Section 2.3.2). The remaining four DOFs of the robotic holder (anteflexion/retroflexion, lateral flexion, multiplane rotation, and handle inclination) operate independently based on direct operator commands through the LabVIEW interface.

The extent of movements for each DOF is determined by the operator through the LabVIEW interface. Commands can be issued within the workspace limits defined in Section 2.1, which are enforced at the motor firmware level. Movement resolution is configured as follows: 0.26° per step for steering wheels, 1° per step for advance/withdraw and turn left/right motions in the leader DOFs, and 3° per step for handle inclination which primarily serves for probe positioning adjustment. Each DYNAMIXEL servomotor incorporates a built-in low-level PID controller to ensure precise execution of operator-defined movements. This incremental control strategy provides fine manipulation resolution while maintaining safe operational speeds appropriate for clinical procedures.

The system utilizes three models of DYNAMIXEL smart servomotors as actuators, each integrating a DC motor, gearbox, encoder, closed-loop controller, and embedded electronics for data acquisition. These actuators communicate through daisy-chained networks using three-wire connections, significantly simplifying the wiring architecture compared to conventional discrete motor-encoder configurations.

**FIGURE 4:**
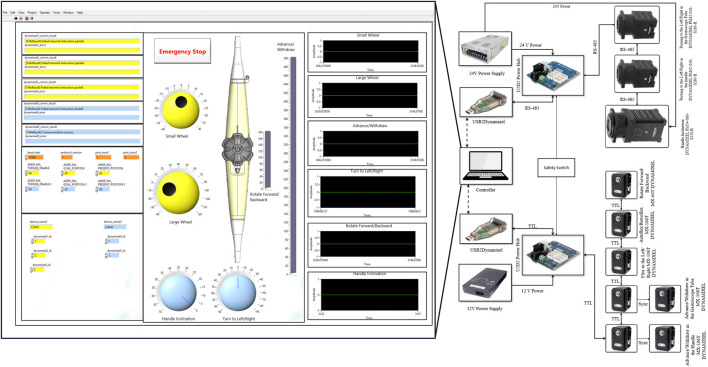
Control architecture and electrical schematic of the robotic system developed in this paper. The LabVIEW user interface (left) provides control panels for all DOFs, position feedback visualization, and emergency stop functionality. The electrical schematic (right) shows the dual-network architecture with TTL-based (12V) and RS-485-based (24V) servomotor networks, USB2Dynamixel interfaces, power distribution hubs, and centralized safety switch integration.

As shown in Figure 4, the system features two distinct actuator networks separated by voltage and communication protocols. The high-torque servomotors (MX-106T and PM-series) operate on 24V power and communicate via RS-485 protocol, while the compact MX-64T servomotors utilize 12V power with TTL-level communication. Both networks interface with the central LabVIEW controller through USB2Dynamixel adapters and incorporate dedicated power distribution hubs (U2D2) for stable operation.

The system incorporates redundant safety mechanisms across hardware and software layers. Emergency stop functionality is implemented both as a physical switch and as a software control within the LabVIEW interface. Motion safety is ensured through constraints in the DYNAMIXEL servomotors that limit both range of motion and velocity. The control system further validates operator inputs, rejecting excessive command changes to maintain gradual and controlled manipulation.

## 3 Kinematic model

Current kinematic modeling approaches for continuum manipulators, including modified Denavit-Hartenberg (DH) methods (Tian et al., 2016; Jones and Walker, 2006) and constant curvature models (Webster III and Jones, 2010; Loschak et al., 2013; Wang et al., 2016a), rely primarily on mechanical principles. While these models can approximate the actual kinematic behavior of the TEE probe, the complex nonlinear behavior of the bending tip leads to significant modeling errors in certain regions of the workspace (Sajadi et al., 2022b). The sources of these nonlinearities can be categorized as follows:

1. Inherent dead zones characterized by hysteresis and back-relaxation in the continuum mechanism at its zero position.
2. The dead zone caused by steering mechanism backlash, which occurs when reversing the direction of the steering wheels.
3. Variable cable-tube friction caused by changing gastroscope tube curvatures.
4. Oversimplified modeling assumptions that neglect plastic torsion and non-constant curvature effects.
5. Nonlinearities resulting from the ovalization of the continuum mechanism’s cross-sectional area.

More sophisticated analytical models could potentially reduce these errors. However, accurately modeling cable friction, hysteresis, and back-relaxation remains computationally intensive and often compromises real-time performance. Our preliminary work (Sajadi et al., 2022b) identified these fundamental limitations, motivating us to pursue a data-driven approach. By combining mechanical design improvements with machine learning, we can capture complex system behavior directly from experimental measurements.

In the following section, we propose a real-time kinematic model utilizing recurrent neural networks (RNNs) with LSTM units, building upon similar approaches in continuum robotics (Tariverdi et al., 2021; Wu et al., 2021; Thuruthel et al., 2019). This method is particularly well-suited for processing time-series data from robotic sensors and effectively models the nonlinear kinematic behavior of our dual-subsystem design. Training on real-world data ensures that all sources of nonlinearity are inherently captured without requiring explicit analytical formulations.

### 3.1 Proposed LSTM-based kinematics model

Recurrent Neural Networks (RNNs) are distinguished from Feed-Forward Networks (FNNs) through their internal feedback loops.This architecture enables RNNs to retain and utilize information from both current and previous time steps, making them particularly effective for time-series data processing. Unlike FNNs, where outputs depend solely on current inputs, RNN outputs are influenced by both present inputs and accumulated internal states. This temporal memory capability allows RNNs to learn complex nonlinear patterns in sequential data, making them ideal for modeling continuum manipulator kinematics with the nonlinearities described in Section 3.

However, standard RNNs suffer from vanishing gradient problems when processing long sequences. Long Short-Term Memory (LSTM) networks address this limitation through specialized gating mechanisms that preserve gradient flow across extended time horizons. LSTMs can capture both short-term dynamics and long-term dependencies in time-varying data, significantly outperforming standard RNNs for complex temporal modeling tasks. The effectiveness of LSTM-based approaches for continuum manipulator modeling has been demonstrated in recent studies (Tariverdi et al., 2021; Wu et al., 2021; Thuruthel et al., 2019).

**FIGURE 5:**
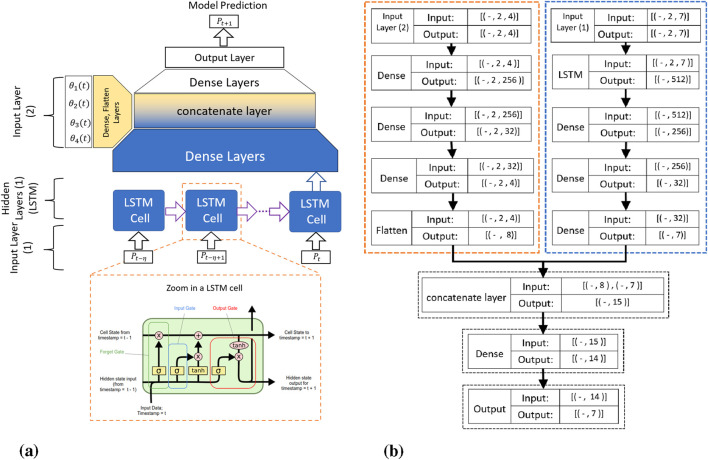
The proposed RNN-based kinematic model: **(a)** Schematic overview showing the time history horizon η , with transducer pose and servomotor commands as inputs, and **(b)** Detailed architecture showing input tensor dimensions, where the first input layer processes TEE transducer pose (Batch Size × 2 × 7) and the second input layer processes servomotor angular positions (Batch Size × 2 × 4).

Figure 5a illustrates our proposed LSTM-based kinematic model architecture. The model processes time-series data through dual input pathways. The first input layer receives the transducer pose vectors p(t) containing position and orientation data:

$$p\left(t\right)={\left[x\left(t\right),y\left(t\right),z\left(t\right),w\left(t\right),v_{1}\left(t\right),v_{2}\left(t\right),v_{3}\left(t\right)\right]}^{T}$$ (4)

where x(t) , y(t) , z(t) represent Cartesian positions and w(t) , $v_{1}\left(t\right)$ , $v_{2}\left(t\right)$ , $v_{3}\left(t\right)$ encode orientation as quaternions. The model utilizes a history horizon η to incorporate temporal context.

The second input layer processes motor command vectors m(t) containing servomotor angular positions:

$$m\left(t\right)=\left[\theta_{1}\left(t\right),\theta_{2}\left(t\right),\theta_{3}\left(t\right),\theta_{4}\left(t\right)\right]$$ (5)

As detailed in Figure 5b, the network architecture consists of parallel processing streams. The pose data flows through LSTM layers (outputting 512-dimensional features) followed by dense layers that progressively reduce dimensionality. The motor commands undergo separate dense layer transformations. Both streams merge at a concatenation layer before final dense layers predict the transducer pose at time t+1 .

Each LSTM cell implements three key gates: the forget gate determines which previous information to discard using sigmoid activation, the input gate selects new information to store by combining sigmoid-gated values with tanh-activated candidate values, and the output gate controls which cell state components contribute to the hidden state output. This gating architecture enables our LSTM-based model to selectively retain relevant temporal patterns while effectively modeling the complex nonlinearities inherent in the TEE probe’s cable-driven mechanism.

## 4 Experiments

Developing our LSTM-based kinematic model requires a comprehensive real-world dataset that captures the full range of system behaviors. This section details our experimental methodology, from sensor deployment and calibration to data acquisition and processing. Our primary objective is to collect diverse TEE probe trajectories throughout the entire workspace, ensuring the dataset includes all nonlinear phenomena identified in Section 3. This comprehensive approach enables rigorous evaluation of the model’s generalization capabilities across varying operating conditions.

### 4.1 Experimental setup

**FIGURE 6:**
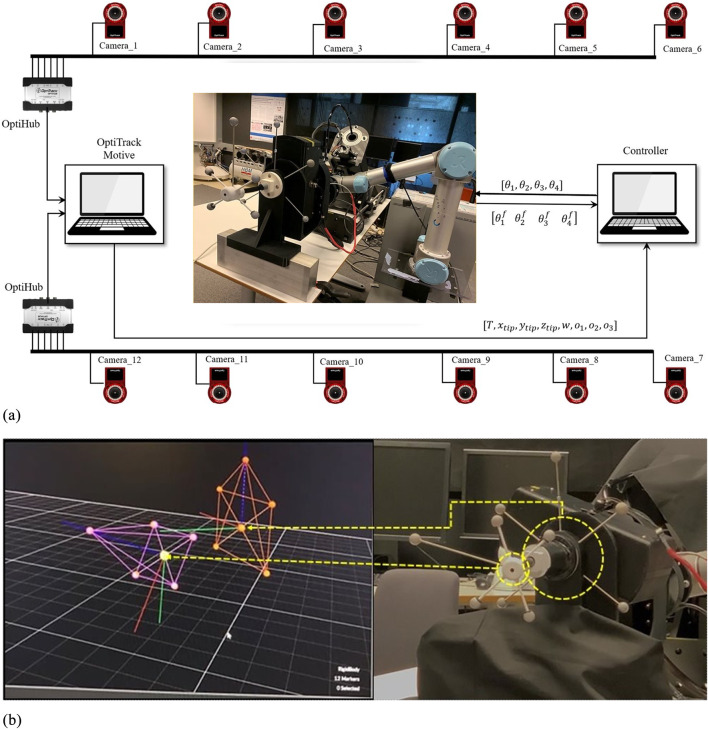
Experimental data acquisition setup. **(a)** OptiTrack visualization of tracked rigid bodies attached to the TEE transducer (purple) and continuum mechanism base (orange). **(b)** System schematic showing 12-camera OptiTrack array, system architecture with motion capture data transmitted via UDP/Ethernet and robot commands sent through TTL/RS-485 protocols, enabling synchronized pose tracking and motor command recording.

Figure 6 illustrates the experimental setup, featuring a 6-DOF OptiTrack optical tracking system with 12 Flex-13 cameras achieving 0.682 mm calibration accuracy. The system simultaneously tracks two key positions: the TEE transducer center at the continuum mechanism tip, and the transition point where the continuum section connects to the gastroscope tube (referred to as the continuum base). Custom 3D-printed rigid bodies, each equipped with five reflective markers, are precisely mounted at these locations. The transducer rigid body’s pivot point aligns with the transducer center, while the second rigid body tracks the continuum base position. Pivot point calibration for both rigid bodies is performed in Motive software, ensuring accurate transformation between marker configurations and actual tracking points.

The OptiTrack Motive software performs 3D rigid body reconstruction and streams 6-DOF pose data (position and orientation) for both tracked points from the Motive PC to the data collection PC via NatNet protocol over UDP/Ethernet, ensuring minimal latency. The data collection PC simultaneously interfaces with the robotic actuators through dedicated serial connections—TTL for low-torque motors and RS-485 for high-torque motors—enabling synchronized recording of motion capture data and motor commands.

### 4.2 Building real-world dataset

Our LSTM-based kinematic model requires comprehensive training data that captures the full spectrum of system behaviors and nonlinearities. Through extensive analysis of TEE probe mechanics and clinical usage patterns, we identified four critical requirements for developing an effective data-driven model:

1. Variable gastroscope tube curvature: During clinical TEE procedures, the gastroscope tube undergoes significant deformation, bending up to 90° as it navigates from the patient’s mouth through the esophagus to the stomach. This varying curvature directly affects the friction between the internal drive cables and the tube’s inner surface, creating position-dependent nonlinearities. Our dataset must encompass the full range of bending angles (0°–90°) to accurately capture these friction-induced variations in system behavior.
2. Continuum mechanism hysteresis: The continuum mechanism at the probe tip exhibits complex hysteretic behavior, particularly when the drive cables relax at the neutral position. This manifests as unpredictable back-relaxation and positional drift, making precise control challenging near the origin. To capture these phenomena, each recorded trajectory includes a deliberate return-to-origin movement, allowing us to observe hysteresis effects under varying approach conditions and cable tensions.
3. Coupled wheel dynamics: Our previous study (Sajadi et al., 2022b) discovered that the large and small steering wheels influence each other’s performance. Specifically, when the large wheel is at certain angles, it changes how much the small wheel slips, and *vice versa*. This mutual interference is worst when either wheel crosses through its zero position (dead zone), where the geometric model’s predictions deviate most from actual behavior. Therefore, our dataset includes trajectories where both wheels operate simultaneously, capturing these coupling effects that single-wheel movements would miss.
4. Comprehensive workspace coverage: To ensure our model generalizes to all possible probe positions during clinical use, the dataset must include movements throughout the full 3D workspace. This means recording trajectories that: (1) translate along all three axes (forward/backward, left/right, up/down), (2) rotate around these axes (pitch, yaw, roll), and (3) combine these movements in various ways. By systematically varying both position and orientation, we ensure the model learns the complete kinematic behavior rather than just specific motion patterns.

**FIGURE 7:**
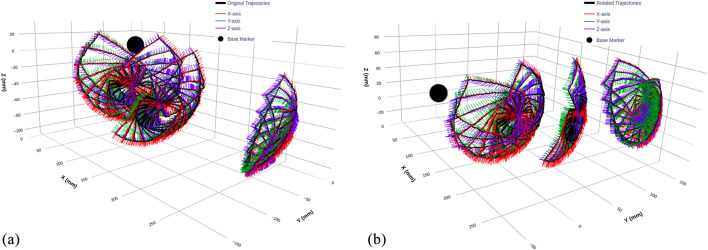
3D visualization of TEE transducer trajectories before and after coordinate transformation. **(a)** Original trajectories recorded by the optical tracking system, displaying raw transducer pose data with overlaid orientation vectors along the X, Y, and Z-axes. **(b)** Transformed trajectories after applying a coordinate alignment with rotation angles of x = 0°, y = −20°, and z = 40°, ensuring a consistent frame of reference across all sub-datasets. The black sphere represents the base of the flexible part of the TEE probe, used as a reference marker.

We selected rectangular trajectories as our fundamental data collection pattern, as illustrated in Figure 7. The rectangular shape is ideal for capturing nonlinearities associated with wheel slip, as it ensures both wheels cross their respective zero positions during each trajectory. Additionally, each trajectory includes a return to the initial position to account for hysteresis and back-relaxation effects in the continuum mechanism, resulting in varying initial conditions for subsequent trajectories. The rectangular pattern also facilitates systematic workspace coverage through geometric transformations.

To map the clinical workspace comprehensively, we collected three sub-datasets corresponding to different gastroscope tube configurations:

- 0° Dataset: Straight tube configuration, representing initial probe insertion.
- 45° Dataset: Moderate bending, typical of mid-esophageal positioning
- 90° Dataset: Maximum curvature, simulating transgastric views

Within each configuration, we systematically rotated trajectories through multiple iterations, ensuring dense workspace sampling while maintaining consistent motion patterns.

#### 4.2.1 Coordinate frame alignment

During experimental setup, the rigid body coordinate frames could not be precisely aligned with the OptiTrack global reference frame due to practical constraints. While the relative motion data remained accurate, this misalignment created uncertainty regarding whether the model would capture true kinematic relationships or develop coordinate-specific dependencies.To address this, we created two dataset versions:

- Original Dataset: Raw trajectory data as captured by the OptiTrack system, shown in Figure 7a
- Rotated Dataset: Coordinate-transformed data (rotation angles: x = 0°, y = −20°, z = 40°) aligned to a standardized world coordinate frame, shown in Figure 7b

Figure 7 clearly illustrates the coordinate transformation effect, with orientation vectors (X, Y, and Z-axes) showing how the rotation realigns the entire trajectory while preserving its geometric structure. The black sphere marks the continuum mechanism base as a consistent reference point across both representations.

**FIGURE 8:**
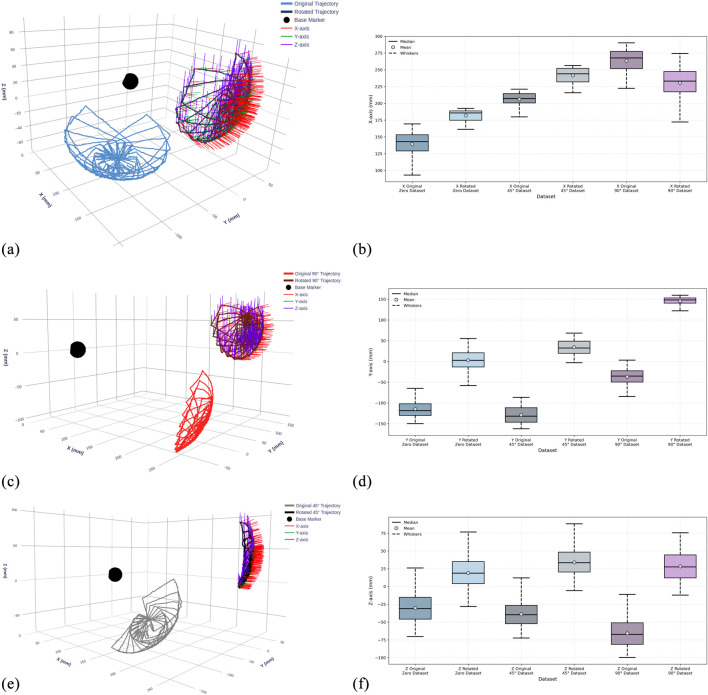
Visualization of recorded TEE transducer trajectories and statistical distributions across three gastroscope tube configurations before and after coordinate frame alignment. **(a,c,e)** 3D trajectory plots showing transducer paths at 0°, 45°, and 90° tube bending angles for the original and rotated dataset. The black sphere indicates the continuum mechanism base. **(b,d,f)** Box plots showing the statistical distribution of transducer positions along X, Y, and Z-axes, comparing data before (original) and after (rotated) coordinate transformation for each configuration.

The two dataset versions provide complementary representations of the same kinematic data in different coordinate frames. Figure 8 visualizes each sub-dataset before and after coordinate transformation: panels (a), (c), and (e) show the 0°, 45°, and 90° datasets respectively. The corresponding box plots in panels (b), (d), and (f) illustrate the statistical distributions along the Y, X, and Z-axes for all three configurations, comparing original and rotated datasets side by side. This coordinate alignment enables rigorous evaluation of model robustness across different reference frames.

Table 1 complements these visualizations with detailed statistical analysis of position values for each dataset before and after rotation, confirming that the transformation preserves motion characteristics while shifting spatial distributions. The availability of both dataset versions enables comprehensive evaluation of the LSTM model’s ability to learn kinematic relationships independent of coordinate frame representation.

**TABLE 1:** Statistical analysis of TEE transducer position data before and after coordinate transformation.

Dataset	Axis	Stage	Position statistics (mm)			
			Mean	Std	Min	Max
Samples: 20,010 — trajectories: 15						
0°Dataset	X	Original	139.34	19.21	85.23	169.42
		Rotated	183.82	11.55	162.48	193.52
	Y	Original	−114.72	20.55	−149.80	−64.93
		Rotated	7.52	14.65	−58.42	56.87
	Z	Original	−30.39	21.20	−70.39	26.06
		Rotated	16.89	22.65	−27.91	76.00
Samples: 14,674 — trajectories: 11						
45°Dataset	X	Original	206.27	10.24	176.13	221.18
		Rotated	241.28	14.04	217.74	256.95
	Y	Original	−128.98	20.40	−162.42	−86.43
		Rotated	34.62	17.66	−2.46	67.13
	Z	Original	−38.95	18.93	−72.35	14.65
		Rotated	34.40	17.27	−6.42	95.45
Samples: 17,342 — trajectories: 13						
90°Dataset	X	Original	263.63	17.30	222.46	290.20
		Rotated	232.02	17.68	171.64	274.29
	Y	Original	−36.65	20.52	−84.60	3.30
		Rotated	148.10	18.73	121.63	150.00
	Z	Original	−65.77	19.09	−99.79	−11.05
		Rotated	28.23	17.02	9.42	74.95

#### 4.2.2 Data acquisition protocol

A custom C++ application managed the synchronized data collection process, sending manipulation commands to the actuators while simultaneously recording 3D poses from the OptiTrack system and angular positions from the servomotor encoders. Data samples were captured at 100 ms intervals, providing sufficient temporal resolution for smooth trajectory reconstruction while ensuring signal stability.The actuation parameters were carefully selected to balance precision with data collection efficiency:

- Steering wheels (MX-106 servomotors): With a resolution of 4096 pulses per revolution, we implemented 3-pulse command increments, achieving an angular precision of 0.26° per step.
- Turn to the Left/Right (PM42-010-S260-R servomotors): Operating at 526,374 pulses per revolution, we applied 21,932-pulse increments (15° steps) to rotate trajectories through multiple angles.

This acquisition protocol generated comprehensive datasets containing 20,010 samples for the 0° configuration, 17,342 samples for 45°, and 14,674 samples for 90°, totaling approximately 52,000 synchronized pose-command pairs. To prevent model bias toward any particular configuration, we downsampled the 0° and 45° datasets to match the smallest set (14,674 samples), ensuring balanced representation across all gastroscope tube configurations during training. This balanced approach provides unbiased training data for our LSTM-based kinematic model.

### 4.3 Training of the proposed LSTM-based kinematics model

The dataset collected from our experimental setup consists of three sub-datasets corresponding to different gastroscope tube configurations: 0° bend containing 20,010 samples, 45° bend with 14,674 samples, and 90° bend comprising 17,342 samples. To avoid training from being biased toward datasets with more samples, we reduced the number of samples in all sub-datasets to 14,000 by extracting continuous segments from each. This ensures equal representation across all gastroscope tube curvatures during training, allowing the model to learn the kinematic behavior uniformly across straight insertion (0°), moderate bending (45°), and maximum curvature (90°) configurations.

#### 4.3.1 Data preprocessing

Prior to training, the raw data underwent preprocessing to prepare it for the neural network. Each sample consists of two synchronized time-series:

- Pose data: $p_{t}={\left[x_{t},y_{t},z_{t},w_{t},v_{1,t},v_{2,t},v_{3,t}\right]}^{T}\in R^{7}$

- Motor commands: $m_{t}={\left[\theta_{1,t},\theta_{2,t},\theta_{3,t},\theta_{4,t}\right]}^{T}\in R^{4}$

The pose data was normalized using zero-mean normalization:

$$p_{\mathrm{norm}}=\frac{p-\mu_{p}}{\sigma_{p}}$$ (6)

where $\mu_{p}$ and $\sigma_{p}$ are the mean and standard deviation computed across each dataset.

#### 4.3.2 Temporal sequence construction

The preprocessed data was partitioned using a 70%-20%–10% split for training, validation, and testing respectively. To capture the temporal dynamics of the cable-driven mechanism, we employed a sliding window approach with history horizon η=20 . For each time step t , the input tensors are constructed as:

Input Layer I (Pose sequences):

$$X_{t}=\left[p_{t-\eta +1},p_{t-\eta +2},\ldots ,p_{t-1},p_{t}\right]\in R^{\eta \times 7}$$ (7)

Input Layer II (Motor sequences):

$$M_{t}=\left[m_{t-\eta +1},m_{t-\eta +2},\ldots ,m_{t-1},m_{t}\right]\in R^{\eta \times 4}$$ (8)

Target output:

$$y_{t}=p_{t+1}\in R^{7}$$ (9)

The sliding window extraction generates input tensors of shape (η×7) for pose sequences and (η×4) for motor commands, providing the temporal context necessary for the LSTM network to model the complex nonlinear dynamics of the cable-driven mechanism.

#### 4.3.3 Model training

The model training was conducted on two versions of the dataset: the original dataset and a coordinate-transformed (rotated) dataset. Both datasets contain identical motion trajectories but in different reference frames, enabling evaluation of the model’s coordinate-frame independence.

For each dataset version, the training followed a cyclical strategy that alternates between all three sub-datasets. Each training cycle consists of one epoch on the 0° sub-dataset, followed by one epoch on the 45° sub-dataset, and one epoch on the 90° sub-dataset. This process was repeated for 20 cycles, resulting in 60 total training epochs distributed equally across all configurations.

The training utilized the Adam optimizer with mean absolute error loss function, a batch size of 32 samples, and enabled data shuffling. This alternating approach ensures balanced learning across all tube configurations without developing bias toward any specific sub-dataset. After completing the training cycles, the final model was evaluated on the test sets for each configuration.

### 4.4 Experimental results

**FIGURE 9:**
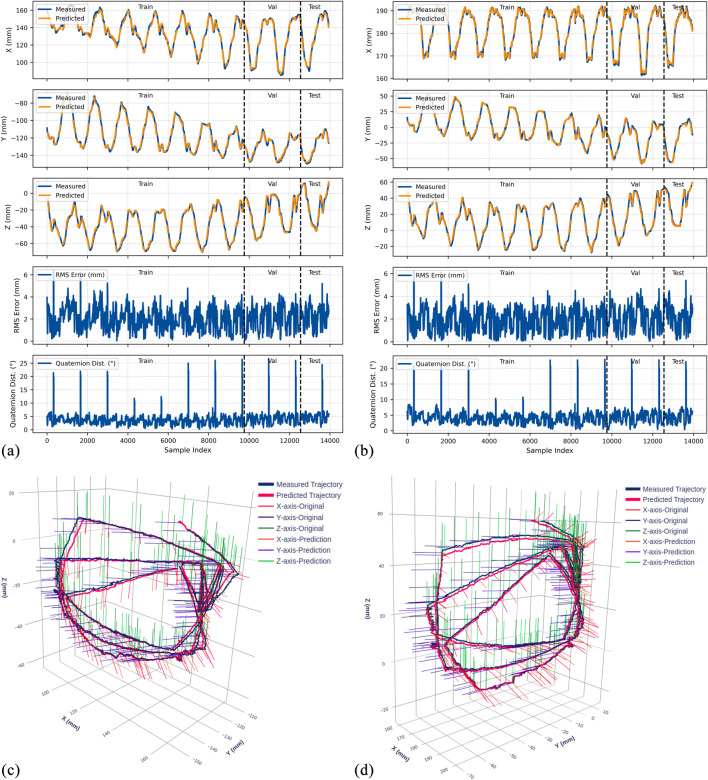
Experimental results for the 0° bend configuration. **(a)** Time-series comparison of recorded (blue) and predicted (orange) trajectories showing X, Y, and Z positions (rows 1–3), RMS position error (row 4), and quaternion distance (row 5) for the original dataset. Vertical dashed lines indicate validation (Val) and test (Test) data boundaries. **(b)** Identical metrics for the rotated dataset. **(c)** 3D trajectory visualization showing recorded data (blue lines) and predicted trajectories (red lines) from validation and test sets, with axes in mm. **(d)** 3D trajectory visualization for the rotated dataset.

**FIGURE 10:**
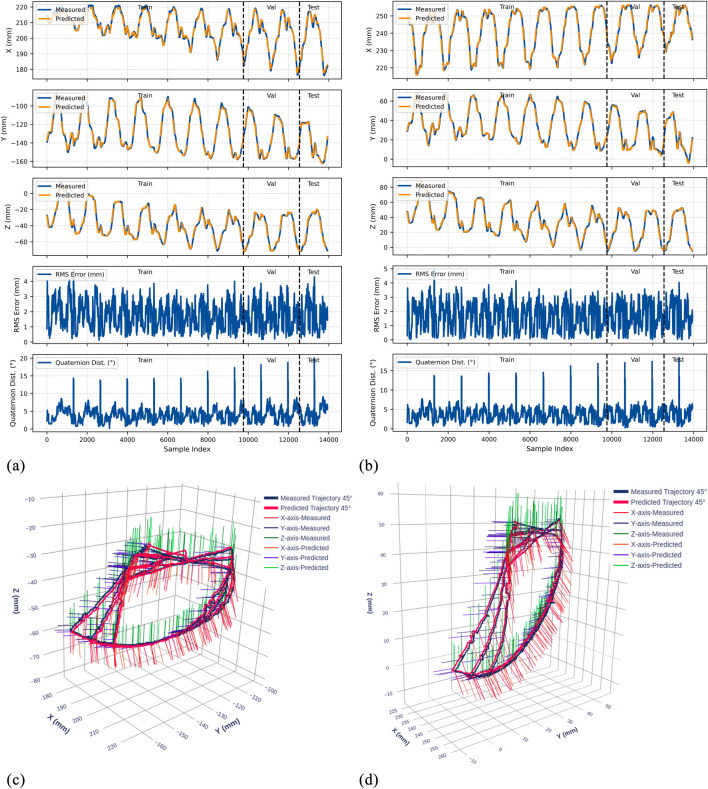
Experimental results for the 45° bend configuration. **(a)** Time-series comparison of recorded (blue) and predicted (orange) trajectories showing X, Y, and Z positions (rows 1–3), RMS position error (row 4), and quaternion distance (row 5) for the original dataset. Vertical dashed lines indicate validation (Val) and test (Test) data boundaries. **(b)** Identical metrics for the rotated dataset. **(c)** 3D trajectory visualization showing recorded data (blue lines) and predicted trajectories (red lines) from validation and test sets, with axes in mm. **(d)** 3D trajectory visualization for the rotated dataset.

**FIGURE 11:**
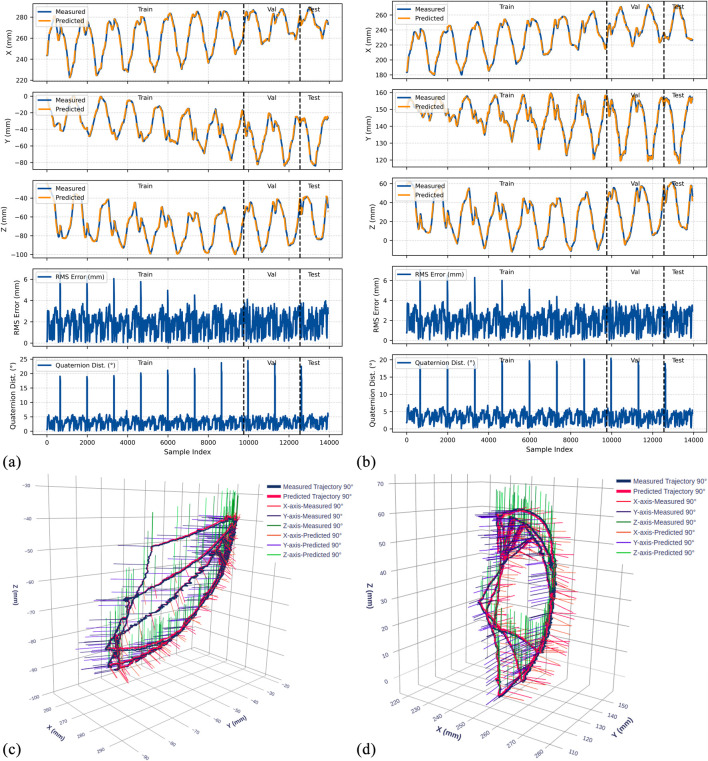
Experimental results for the 90° bend configuration. **(a)** Time-series comparison of recorded (blue) and predicted (orange) trajectories showing X, Y, and Z positions (rows 1–3), RMS position error (row 4), and quaternion distance (row 5) for the original dataset. Vertical dashed lines indicate validation (Val) and test (Test) data boundaries. **(b)** Identical metrics for the rotated dataset. **(c)** 3D trajectory visualization showing recorded data (blue lines) and predicted trajectories (red lines) from validation and test sets, with axes in mm. **(d)** 3D trajectory visualization for the rotated dataset.

The trained LSTM-based kinematic model was evaluated on both original and rotated datasets across all three gastroscope tube configurations (0°, 45°, and 90° bends). Figures 9–11 present comprehensive evaluation results for each configuration, with detailed quantitative metrics summarized in Table 2. The position metrics were calculated by comparing the predicted and recorded dataset as follow:

$$\text{MSE}=\frac{1}{n}\overset{n}{\underset{i=1}{\sum }}{\left(p_{i}-{\hat{p}}_{i}\right)}^{2}$$ (10)

$$\text{RMSE}=\sqrt{\text{MSE}}$$ (11)

$$R^{2}=1-\frac{\sum_{i=1}^{n}{\left(p_{i}-{\hat{p}}_{i}\right)}^{2}}{\sum_{i=1}^{n}{\left(p_{i}-\bar{p}\right)}^{2}}$$ (12)

where $p_{i}$ and ${\hat{p}}_{i}$ represent the recorded and predicted position vectors, respectively, and $\bar{p}$ is the mean position.

**TABLE 2:** Performance metrics of the LSTM-based kinematic model across different tube configurations for both original and rotated datasets.

Dataset	Sub-dataset	Split	Position metrics			Orientation metrics	
			MSE ( ${\text{mm}}^{2}$ )	RMSE (mm)	R^2^	Mean (°)	RMSE (°)
Original	0°	Train	0.0130	1.140	0.9842	8.936	11.344
		Val	0.0134	1.157	0.9827	8.609	9.777
		Test	**0.0161**	**1.267**	**0.9818**	**7.064**	**7.690**
	45°	Train	0.0134	1.159	0.9832	11.908	13.842
		Val	0.0132	1.148	0.9869	10.889	12.182
		Test	**0.0146**	**1.209**	**0.9861**	**8.503**	**9.223**
	90°	Train	0.0121	1.098	0.9874	8.017	10.055
		Val	0.0123	1.110	0.9830	6.351	7.877
		Test	**0.0143**	**1.194**	**0.9782**	**4.947**	**5.855**
Rotated	0°	Train	0.0122	1.104	0.9856	10.924	12.969
		Val	0.0170	1.305	0.9840	10.608	11.732
		Test	**0.0186**	**1.364**	**0.9808**	**9.085**	**9.909**
	45°	Train	0.0121	1.099	0.9876	11.548	13.263
		Val	0.0111	1.055	0.9875	10.334	11.772
		Test	**0.0119**	**1.092**	**0.9849**	**8.085**	**8.790**
	90°	Train	0.0137	1.170	0.9838	9.521	11.661
		Val	0.0182	1.350	0.9808	7.472	8.480
		Test	**0.0194**	**1.392**	**0.9811**	**5.793**	**6.677**

Position metrics represent the average across X, Y, and Z-axes. Test results are highlighted in gray. Bold values indicate test set results, representing the model's performance on unseen data.

The orientation data accuracy was evaluated using the quaternion angular distance:

$$\theta_{\text{error}}=2\arccos\left(\left|q_{\text{recorded}}\cdot q_{\text{predicted}}\right|\right)$$ (13)

where $q_{\text{recorded}}$ and $q_{\text{predicted}}$ are the normalized quaternions. This metric provides the rotational error in degrees.

#### 4.4.1 Position prediction accuracy

The model demonstrates strong position tracking with RMSE values ranging from 1.092 mm to 1.392 mm and R^2^ values above 0.978 across all test datasets. Time-series plots (Figures 9–11, panels a–b) show accurate trajectory capture for all sub-datasets, with predicted trajectories closely following recorded data. RMS error plots reveal consistent baseline performance with characteristic spikes occurring only at trajectory transitions, where the transducer returns to its initial position before beginning the next trajectory. These transient errors rapidly return to baseline levels, confirming the model’s robustness in maintaining tracking accuracy.

#### 4.4.2 Orientation prediction performance

The orientation tracking results reveal interesting configuration-dependent behavior. While the 0° and 45° configurations show relatively similar performance with mean orientation errors of 7.064° and 8.503° respectively (and RMSE values of 7.690° and 9.223°), the 90° configuration demonstrates markedly superior results with a mean error of only 4.947° and RMSE of 5.855°. Notably, the 90° bend configuration represents the clinically relevant workspace where the transducer is positioned within the esophagus for cardiac imaging—our primary area of interest.

The quaternion distance plots (row 5 in Figures 9–11) demonstrate that orientation errors remain relatively stable throughout the trajectories, with occasional spikes coinciding with trajectory transitions at the beginning of each new path.

#### 4.4.3 Coordinate frame independence

The performance comparison between original and rotated datasets validates the model’s coordinate frame independence. Analysis of the original and rotated datasets reveals consistent performance: the average test RMSE across all configurations is 1.223 mm for the original dataset and 1.283 mm for the rotated dataset, a difference of only 0.06 mm. For orientation metrics, the average difference is 1.02°.

These small variations confirm that the LSTM network captures the underlying kinematic relationships rather than coordinate-specific patterns. The 3D trajectory visualizations in Figures 9–11 (panels c and d) further support this finding. Such coordinate independence proves essential for practical deployment, ensuring model accuracy remains consistent despite reference frame changes.

## 5 Discussion

This study presents a robotic-assisted TEE system that builds upon the dual-subsystem architecture pioneered and clinically validated by Schewel et al. (2024), Schewel et al. (2025), while introducing critical enhancements in robotic system design and deep-learning based kinematic modeling. Previous systems (Wang et al., 2016a; Wang et al., 2016b; Xie et al., 2023; Sajadi et al., 2022b) manipulate the TEE probe exclusively from the handle, limiting their ability to replicate manual TEE by echocardiographer. Schewel et al. pioneered the dual-subsystem approach by demonstrating simultaneous manipulation from both the handle and gastroscope tube. Their successful first-in-human trials established the clinical feasibility of this architecture.

Building upon Schewel’s validated concept, we developed a robotic assisted TEE system with enhanced flexibility through integration with a UR5 collaborative manipulator and extra DOF on the TEE robot. Our system incorporates all degrees of freedom from the ROB’E design while adding critical capabilities for broader clinical implementation. The developed system in this paper features 6 DOF at the probe handle (robotic holder subsystem) and 2 DOF at the gastroscope tube (add-on robotic subsystem), with the UR5 integration providing dynamic repositioning capabilities.

A key limitation of Schewel’s ROB’E system is its fixed gastroscope tube control mechanism positioned at the head of the operating table. This configuration works well for supine positioning typically used during interventional procedures. However, it cannot accommodate left lateral decubitus positioning, which is the standard orientation for awake patients undergoing diagnostic TEE with conscious sedation. Our system addresses this limitation through integration with the UR5 manipulator, which enables robotic repositioning of the add-on robotic subsystem to accommodate both supine and left lateral decubitus patient orientations. This adaptability extends the system’s clinical utility from interventional procedures, where patients are typically intubated in supine position, to diagnostic examinations performed on conscious patients in left lateral positioning.

Furthermore, our system incorporates two additional degrees of freedom absent in the ROB’E design. The first is a handle inclination DOF (0°–70° range) that replicates the standard manual TEE holding technique documented in clinical guidelines (Shanewise et al., 1999; Hahn et al., 2013; Jaidka et al., 2019). The second is rail-mounted advance/withdraw capability at the handle position. Together, these enhancements create a gentle, gradual curvature in the gastroscope tube, in contrast to the sharp U-bend that occurs with ROB’E’s fixed mounting. This optimized geometry significantly reduces cable tension asymmetry between inner and outer radius cables, while also decreasing friction between the cables and the inner surface of the gastroscope tube. By minimizing these friction-induced nonlinearities and dead zones, the developed robotic system creates predictable kinematic behavior suitable for data-driven kinematic modeling. The optimized geometry not only facilitates accurate kinematic modeling but also reduces mechanical stress on the expensive TEE probe during prolonged procedures. This approach preserves equipment lifespan comparable to manual operation while ensuring consistent and reliable robotic control.

The primary contribution of this work lies in successfully addressing the complex kinematic modeling challenge through deep learning integration. Conventional analytical approaches, including modified Denavit-Hartenberg methods (Jones and Walker, 2006; Tian et al., 2016) and constant curvature models (Webster III and Jones, 2010; Loschak et al., 2013; Wang et al., 2016a), rely on simplified geometric assumptions that fail to capture the true behavior of cable-driven continuum mechanisms. As demonstrated in our previous work (Sajadi et al., 2022b), conventional methods struggle with inherent dead zones at zero positions, hysteresis from cable relaxation, and backlash in the steering mechanisms. These challenges intensify when both steering mechanisms are manipulated simultaneously, creating significant modeling errors that compromise real-time control accuracy of continuum manipulators.

The developed LSTM-based kinematic model inherently addresses these nonlinearities through its temporal memory mechanism. The LSTM architecture captures history-dependent behavior without requiring explicit hysteresis elimination algorithms. By training on trajectories that deliberately manipulate both steering wheels simultaneously and include return-to-origin movements, the model learns the complex patterns of hysteresis, backlash, and coupling effects characteristic of cable-driven continuum mechanism. This enables effective mapping of dead zone transitions and cable relaxation effects. The model’s ability to maintain tracking accuracy (RMSE < 1.4 mm) throughout the workspace, despite these inherent nonlinearities, validates the effectiveness of our data-driven approach for real-time modeling of continuum manipulator.

The model was trained on 42,000 synchronized pose-command pairs across three gastroscope tube configurations (0°, 45°, 90° bends). This comprehensive dataset demonstrates that complex cable-driven nonlinearities can be effectively captured through data-driven approaches. Position tracking achieves RMSE below 1.4 mm, exceeding precision requirements for cardiac structures. These structures range from 2–4 mm coronary arteries to 20–25 mm diameter aortic valves.

The 90° bend configuration showed superior performance with mean orientation error of 4.947° and RMSE of 5.855°. This configuration represents typical esophageal curvature during clinical procedures and corresponds to the primary workspace for transgastric and deep esophageal views. These accuracy levels enable reliable visualization for TEER and paravalvular leak closure procedures. Given the 25–35 mm mitral valve annulus dimensions, our positioning error becomes negligible for clinical decision-making.

The model demonstrates coordinate frame independence with only 0.06 mm RMSE difference between original and rotated datasets. This confirms true kinematic learning rather than coordinate-specific pattern memorization. These results validate the model’s effectiveness in handling the diverse nonlinearities present in cable-driven TEE mechanisms while maintaining sub-millimeter accuracy. The system achieves real-time performance with 1.8 ms inference time, meeting the computational requirements for clinical implementation.

The successful integration of mechanical design improvements with data-driven kinematic modeling establishes a pathway toward semi-autonomous TEE systems. The demonstrated accuracy and real-time performance provide a solid foundation for future developments, including integration with deep learning-based image analysis for automated view optimization and real-time anatomical structure recognition. As cardiovascular interventions continue evolving toward less invasive approaches and the demand for specialized imaging expertise grows, robotic systems that can operate both locally and remotely will become increasingly vital for delivering high-quality cardiac care across diverse clinical settings.

### 5.1 Limitations and future work

Although the developed system achieves high positioning accuracy in controlled laboratory settings, validation must progress systematically through increasingly realistic scenarios. The next phase involves testing with anatomically accurate esophageal phantoms that replicate tissue properties and geometric constraints. Following successful phantom validation, animal studies will evaluate system performance in dynamic physiological conditions including cardiac motion, respiratory effects, and peristalsis. These preclinical studies will inform safety protocols and operator training requirements before proceeding to first-in-human trials.

Another limitation of the current system is the absence of force feedback and compliance control mechanisms. Safe interaction with esophageal tissues requires real-time force monitoring to prevent tissue trauma during probe manipulation. Future iterations must incorporate force/torque sensors at both the probe-tissue interface and along the gastroscope tube to detect excessive contact forces. Ning protocols, remain necessary before human implementation.

The current teleoperation framework through LabVIEW provides a foundation for advancing toward semi-autonomous capabilities. Future development will focus on integrating deep learning-based image analysis for automated view recognition and optimization. This could enable features such as automatic acquisition of standard TEE views, real-time anatomical structure identification, and guided navigation to target cardiac structures. The demonstrated kinematic accuracy and real-time performance of our LSTM model provide the control precision necessary for these advanced autonomous functions.

The developed robotic system builds on commercially available TEE probes without any modifications, which simplifies regulatory pathways by treating the robot as an external manipulation device rather than modifying the FDA-approved probe itself. This approach, combined with our demonstrated accuracy and reliability, positions the system for accelerated clinical translation. However, comprehensive safety testing in phantom and animal trials, followed by clinical trials and operator training protocols, remain necessary before human implementation.

## 6 Conclusion

This paper presented a robotic-assisted transesophageal echocardiography (TEE) system that extends the dual-subsystem architecture established by Schewel et al. through robotic system design enhancements and deep learning modeling. The system incorporates 6 DOF at the probe handle and 2 DOF at the gastroscope tube, fully replicating manual TEE procedures performed by echocardiographers. Integration with our previously developed teleoperated UR5 system enables dynamic repositioning of the gastroscope tube control mechanism to accommodate both supine and left lateral decubitus patient positions. Two additional DOFs, handle inclination and rail-mounted advance/withdraw, create optimal gastroscope tube geometry that minimizes cable-driven nonlinearities while maintaining the natural manipulation technique of clinical practice.

The primary contribution is the LSTM-based kinematic model trained on 42,000 synchronized pose-command pairs collected directly from our robotic system across three gastroscope tube configurations. The model achieves position RMSE below 1.4 mm and orientation error of 4.947° at the clinically relevant 90° bend configuration. The model’s robustness is demonstrated through coordinate frame independence testing, with only 0.06 mm RMSE difference between original and rotated datasets, confirming true kinematic learning rather than coordinate-specific patterns. With 1.8 ms inference time, the model demonstrates real-time performance essential for responsive robotic control.

This work demonstrates that a robotic system incorporating all clinical DOFs, combined with data-driven modeling, can effectively address the complex nonlinearities inherent in cable-driven TEE mechanisms. The current teleoperation capability through LabVIEW provides immediate clinical utility while the LSTM-based kinematic modeling establishes a foundation for semi-autonomous TEE systems. Future development will focus on force feedback integration, clinical validation, and incorporation of deep learning-based image analysis for automated view optimization and procedural guidance.

## Data Availability

The raw data supporting the conclusions of this article will be made available by the authors, without undue reservation. The code and trained models are publicly available at: https://github.com/GandalfTech/robotic-TEE-LSTM-kinematics. Additional raw data will be made available upon request.
